# Erlotinib treatment after platinum-based therapy in elderly patients with non-small-cell lung cancer in routine clinical practice – results from the ElderTac study

**DOI:** 10.1186/s12885-018-4208-x

**Published:** 2018-03-27

**Authors:** Wolfgang M. Brueckl, H. Jost Achenbach, Joachim H. Ficker, Wolfgang Schuette

**Affiliations:** 1 Department of Respiratory Medicine, Allergology and Sleep Medicine, Paracelsus Medical University Nuernberg, General Hospital Nuernberg, Prof.-Ernst-Nathan-Str. 1, Nuremberg, Germany; 2 Lung Clinic Lostau, Department of Thoracic Oncology, Lindenstr. 2, Lostau, Nuremberg, Germany; 3 Hospital Martha-Maria Halle-Doelau, Klinik für Innere Medizin II, Röntgenstr. 1, Halle, Germany

**Keywords:** Aged, Epidermal growth factor receptor, Non-small-cell lung carcinoma, Second line, Tyrosine kinase inhibitor

## Abstract

**Background:**

In this prospective non-interventional study, the effectiveness and tolerability of erlotinib in elderly patients with non-small-cell lung cancer (NSCLC) after ≥1 platinum-based chemotherapy were assessed.

**Methods:**

A total of 385 patients ≥65 years of age with advanced NSCLC receiving erlotinib were observed over 12 months. The primary endpoint was the 1-year overall survival (OS) rate.

**Results:**

Patients were predominantly Caucasian (99.2%), a mean of 73 years old; 24.7% had an Eastern Cooperative Oncology Group performance status (ECOG PS) ≥2. Most common tumor histologies were adenocarcinoma (64.9%) and squamous cell carcinoma (22.3%). Of 119 patients tested, 15.1% had an activating epidermal growth factor receptor gene (*EGFR)* mutation. The 1-year OS rate was 31% (95% CI 25–36) with a median OS of 7.1 months (95% CI 6.0–7.9). OS was significantly better in females than males (*p* = 0.0258) and in patients with an *EGFR* mutation compared to *EGFR* wild-type patients (*p* = 0.0004). OS was not affected by age (*p* = 0.3436) and ECOG PS (*p* = 0.5364). Patients with squamous NSCLC tended to live longer than patients with non-squamous *EGFR* wild-type tumors (median OS: 8.6 vs 5.5 months). Cough and dyspnea improved during the observation period. The erlotinib safety profile was comparable to that in previous studies with rash (45.2%) and diarrhea (22.6%) being the most frequently reported adverse events.

**Conclusions:**

Erlotinib represents a suitable palliative treatment option in further therapy lines for elderly patients with advanced NSCLC. The results obtained under real-life conditions add to our understanding of the benefits and risks of erlotinib in routine clinical practice.

**Trial registration:**

BfArM (https://www.bfarm.de; ML23023); ClinicalTrials.gov (NCT01535729; 20 Feb 2012).

## Background

Lung cancer is the leading cause of cancer deaths worldwide [1]; about 85% of cases are diagnosed as non-small-cell lung cancer (NSCLC) [2]. The median age of NSCLC patients is 70 years and the disease is usually diagnosed in advanced stages, when curative surgery is no longer feasible [3]. In metastasized disease, first-line chemotherapy is often not successful and the 5-year survival rate is only 4.2% [3]. NSCLC is histologically classified into the major subtypes adenocarcinoma (~ 40%) [4, 5], squamous cell carcinoma (~ 30–40%) [6–9] and large cell carcinoma (~ 5–10%) [9]. Survival has improved for all subtypes in recent years, but the extent of improvement has been higher for adenocarcinoma than squamous tumors [10]. Recurring mutations have been reported in genes coding for epidermal growth factor receptors (EGFR) in 10–40% of adenocarcinomas [11–13], but these mutations are rare in squamous tumors [14]. *EGFR* mutations can lead to constitutive activation of anti-apoptotic and proliferation signaling pathways, which promote cancer progression [15].

EGFR tyrosine kinase inhibitors (TKI) are the preferred first-line treatment for advanced NSCLC with *EGFR* mutations [16, 17], and the EGFR-TKI erlotinib (Roche Pharma, Tarceva®, Basel, Switzerland) is also approved in Europe for treatment of patients with *EGFR* wild-type tumors after failure of at least one prior chemotherapy regimen [18].

Treating NSCLC is challenging because of the advanced age of patients. As EGFR-TKI avoid the systemic side effects of traditional chemotherapy they might be more suitable for treating elderly patients [19]. A large phase-3 trial with erlotinib including 586 younger and 163 elderly patients demonstrated a similar survival and quality of life (QoL) in both age groups, although a somewhat higher toxicity in the elderly was observed [20]. Clinical studies examining the elderly population are limited and often firm conclusions cannot be drawn [21, 22]. In this study (ElderTac: erlotinib in routine clinical practice in elderly patients with NSCLC), we examined the effectiveness and tolerability of erlotinib in elderly NSCLC patients with progressive disease on ≥1 platinum-based chemotherapy in Germany.

## Methods

### Study design

ElderTac was a multicenter, non-comparative, non-interventional, single-arm surveillance study documenting erlotinib treatment during routine clinical practice in Germany between April 2011 and August 2014. The observation period was 12 months. Information was gathered during examinations by the physician at baseline and after 3, 6, 9, and 12 months.

This study was conducted in accordance with the German Medicines Act (AMG chapter 67, section 6). It was registered with the German Federal Institute for Drugs and Medical Devices (BfArM) and at ClinicalTrials.gov (NCT01535729). Regular monitoring of study documentation in every center was performed by AMS Advanced Medical Services GmbH, Mannheim, Germany.

### Patients and treatment

Elderly patients (≥65 years) with advanced or metastatic UICC stage IV NSCLC, confirmed by histological analysis, were recruited. Histological and immunohistochemical analysis was used to distinguish different types of NSCLC. Patients were eligible if they had progressive disease on ≥1 platinum-based chemotherapy treatment. Erlotinib was prescribed to patients in accordance with the terms of the marketing authorization. Specific treatment and diagnostic procedures were at the discretion of the treating physician.

### Outcome measurements

The main outcome parameter was the 1-year overall survival (OS) rate. In addition, OS, 1-year progression-free survival (PFS) rate, PFS, objective response rate (ORR), disease control rate (DCR), symptom control, and adverse events (AE) were assessed. The ORR was defined as the proportion of patients with at least a partial response. The DCR was defined as the complete response + partial response + stable disease. Response to treatment was assessed by the investigator using RECIST criteria (version 1.1). AEs were coded by the Medical Dictionary for Regulatory Activities (MedDRA) (version 15.1).

### EGFR mutation status

As erlotinib is approved in Europe for second−/third-line therapy of metastatic NSCLC irrespective of *EGFR* mutation status [18], *EGFR* mutation testing was performed at the discretion of the participating centers. *EGFR* testing using sequencing strategies was done by certified molecular pathology departments collaborating with the individual study centers. Results were documented as: not tested, not available, *EGFR* activating mutation or wild-type.

### Statistics

To accurately estimate the 1-year OS, 400 patients were considered necessary, assuming a survival rate of 33 ± 4.6%, and using a symmetric 95% confidence interval (CI, calculated using Greenwood’s standard error estimate). The survival rate was estimated to be 33% based on publications of four big international studies [23–26]. Other data were analyzed descriptively.

The effectiveness and safety for all patients who received ≥1 dose of erlotinib were analyzed. Continuous and categorical data were described as median (minimum, maximum) and frequencies/percentages, respectively.

Survival was analyzed by Kaplan Meier methodology and survival curves were compared using an unstratified log-rank test. Survival and response data were analyzed overall and in the following subgroups: age (65–69, 70–74, 75–79, ≥80 years or < 75 and ≥ 75 years), *EGFR* mutation (positive or wild type), Eastern Cooperative Oncology Group performance status (ECOG PS) (0, 1, ≥2) and gender. The influence of age, gender and *EGFR* mutation status on the OS was additionally investigated using Cox regression models (considering single and multiple factors). Post-hoc analysis was performed to compare younger with older patients (< 75 or ≥ 75 years) and non-squamous *EGFR* wild-type carcinoma with squamous carcinoma.

No correction for missing data was performed.

## Results

### Patients

In 102 centers, 465 patients were screened for eligibility. Eighty patients were excluded for the following reasons: no previous failed platinum-based chemotherapy (33), NSCLC UICC stage IV histology not confirmed (20), < 65 years old (11), erlotinib not administered (9), patient records unavailable (5), lack of informed consent (1) and screening failure (1). In total, 385 patients were included in the analysis. At 3 months, data were available for 380 patients (98.7%). This decreased to 159 patients (41.3%) at 6 months, 80 (20.8%) at 9 months, and 54 (14.0%) at 12 months. The main reason for discontinuation was disease progression (60% patients).

The patients’ baseline data are presented in Table 1. The median age was 72 years (range: 62–90 years). The most common tumor histology was adenocarcinoma (64.9%), followed by squamous cell carcinoma (22.3%), and large cell carcinoma (4.2%). *EGFR* mutation screening was performed in 31% of patients and 15.1% had a positive *EGFR* mutation status. Although *EGFR* mutation testing was mainly performed in patients with adenocarcinoma, other histological tumor types cannot be excluded. Thus, we refer to non-squamous *EGFR* wild-type carcinoma hereafter.

**Table 1:** Patient baseline characteristics (*N* = 385)

Patient characteristics		Patients, *n* (%)
Age, years	65–69	110 (28.6)
	70–74	140 (36.4)
	75–79	94 (24.4)
	≥ 80	37 (9.6)
	NR	4 (1.0)
Gender	Male	258 (67)
	Female	127 (33)
Ethnicity	Caucasian	382 (99.2)
	Asian	2 (0.5)
	Afro-American	0 (0)
	Other	1 (0.3)
ECOG PS	0	64 (16.6)
	1	210 (54.5)
	2	92 (23.9)
	3	3 (0.8)
	NR	16 (4.2)
Smoking status	Never smoked	91 (23.7)
	Former smoker	198 (51.4)
	Current smoker	77 (20.0)
	NR	19 (4.9)
Tumor histology	Adenocarcinoma	250 (64.9)
	Squamous cell carcinoma	86 (22.3)
	Large cell carcinoma	16 (4.2)
	Bronchoalveolar carcinoma	11 (2.9)
	Adenoid squamous cell carcinoma	10 (2.6)
	Other	12 (3.1)
*EGFR* mutation status	Tested	119 (30.9)
	Positive	18 (15.1)
	Wild-type	98 (82.4)
	Indefinite	3 (2.5)
Previous chemotherapy	Carboplatin	279 (72.5)
	Cisplatin	124 (32.2)
	Docetaxel	54 (14.0)
	Gemcitabine	96 (24.9)
	Paclitaxel	56 (14.5)
	Vinorelbine	92 (23.9)
	Other	182 (47.3)

*EGFR* epidermal growth factor receptor gene, *ECOG PS* Eastern Cooperative Oncology Group performance status, *NR* not recorded

At baseline, 16.6% and 54.5% of patients had an ECOG PS of 0 or 1, respectively, while 24.7% had an ECOG PS ≥2. This did change slightly at the 3-month visit, where 9.1% and 35.6% had an ECOG PS of 0 or 1, respectively. The percentage of patients with an ECOG PS ≥3 was < 3% for the rest of the observation period. The majority of patients had concomitant diseases (86.2%). The main comorbid conditions were chronic obstructive pulmonary disease (33.0%), diabetes mellitus (21.6%), heart failure (8.1%), coronary heart disease/angina pectoris (14.0%), peripheral arterial occlusion disease (10.4%), and stroke (6.8%).

All patients had previously received chemotherapy, mainly based on carboplatin (72.5%) and/or cisplatin (32.2%) (Table 1). Six or more cycles of chemotherapy were completed in 43.3% of patients, and only one cycle was completed in 4.5% of patients. Radiotherapy had been previously administered to 35.8% of patients. Additionally, 24.9% of patients had received previous surgical treatment; 43.8% of these with curative intent.

### Treatment

At baseline, 91.7% of patients received the recommended daily dose of 150 mg erlotinib. Erlotinib dose was modified during the study course as follows: 3/6 months: increased in 4/3 patients (1.1/1.9%), reduced in 32/11 patients (8.7/7.1%), interrupted in 20/10 patients (5.4/6.5%), and discontinued in 192/64 patients (35/41.6%) out of 368/154 remaining patients. The main reason for dose reduction was intolerance (3/6 months: 27/6 patients [7.3/3.9%]). The main reason for discontinuation was disease progression (3/6 months: 132/49 patients [35.8/31.8%]).

### Effectiveness of erlotinib treatment in elderly patients

#### Treatment response

Six months after treatment onset in the overall population, 2 of the 127 patients evaluated (1.6%) had a complete response, 12 (9.4%) had a partial response and 55 (43.3%) had stable disease. In *EGFR* wild-type patients, 1 of the 22 evaluated (3.6%) had a complete response, 1 (3.6%) had a partial response and 9 (40.9%) had stable disease after six months. ORR and DCR at the 3-month and the 6-month visit and treatment responses stratified according to tumor histology are displayed in Table 2.

**Table 2:** Clinical endpoints stratified by patient baseline characteristics for the overall population, in patients with squamous NSCLC, and in patients with non-squamous *EGFR* wild-type tumors

	ORR 3/6 months (%)	DCR 3/6 months (%)	Median PFS (months)	Median OS (months)	1-year OS (%)
All patients (*N* = 385)					
Overall	5.7/3.6	30.9/17.9	3.5	7.1	30.6
Age (years)					
65–69	4.5/1.8	25.5/12.7	3.3	7.0	26.6
70–74	4.3/2.9	27.1/18.6	3.4	6.6	29.8
75–79	7.4/6.4	42.6/23.4	5.0	7.9	37.2
≥ 80	10.8/5.4	32.4/16.2	2.9	6.0	25.6
< 75^a^ (*n* = 250)	4.4/2.4	26.4/16.0	3.3	6.8	28.3
≥ 75^a^ (*n* = 131)	8.4/6.1	39.7/21.4	4.0	7.8	34
ECOG PS					
0	6.3/3.1	28.1/17.2	3.3	8.4	37.6
1	6.2/3.3	31.4/16.7	3.5	6.3	29.9
≥ 2	4.2/5.3	33.7/23.2	3.7	7.3	29.1
Gender					
Male	5.8/2.7	31.8/16.7	3.4	6.3	25.4
Female	5.5/5.5	29.1/20.5	4.1	8.1	41.8
Post-hoc analysis: Squamous cell histology (*n* = 86)					
Overall	8.1/1.2	34.9/18.6	3.5	8.6	32.4
Age (years)					
< 75 (*n* = 55)	5.5/1.8	29.1/18.2	3.5	9.5	33.1
≥ 75 (*n* = 30)	13.3/0	43.3/16.7	3.6	7.8	29.1
ECOG PS					
0 (*n* = 12)	8.3/0	50.0/16.7	4.6	8.7	31.4
1 (*n* = 44)	11.4/0	34.1/20.5	3.7	11.2	43.3
≥ 2 (*n* = 27)	3.7/3.7	33.3/18.5	3.5	7.3	19.2
Gender					
Male (*n* = 66)	6.1/1.5	33.3/16.7	3.5	8.6	28.7
Female (*n* = 20)	15.0/0	40.0/25.0	4.4	9.5	44.5
Post-hoc analysis: Non-squamous *EGFR* wild-type (*n* = 91)					
Overall	3.3/2.2	20.9/11.0	3.1	5.5	28.8
Age (years)					
< 75 (*n* = 60)	1.7/0	20.0/8.3	3.1	5.2	22.3
≥ 75 (n = 30)	6.7/6.7	23.3/16.7	3.2	7.9	41.7
ECOG PS					
0 (*n* = 18)	5.6/0	22.5/5.6	2.2	7.0	30.9
1 (*n* = 50)	2.0/2.0	20.0/10.0	2.6	5.5	23.7
≥ 2 (n = 18)	0/5.6	22.2/22.2	4.2	6.8	39.2
Gender					
Male (*n* = 54)	1.9/0	18.5/5.6	2.1	4.7	16.2
Female (*n* = 37)	5.4/5.4	24.3/18.9	5.0	9.3	47.9

^a^post-hoc analysis

*EGFR* epidermal growth factor receptor gene, *NSCLC* non-small-cell lung cancer, *OS* overall survival, *PFS* progression-free survival, *ORR* objective response rate, *DCR* disease control rate, *ECOG PS* Eastern Cooperative Oncology Group performance status

#### Survival

**Fig. 1:**
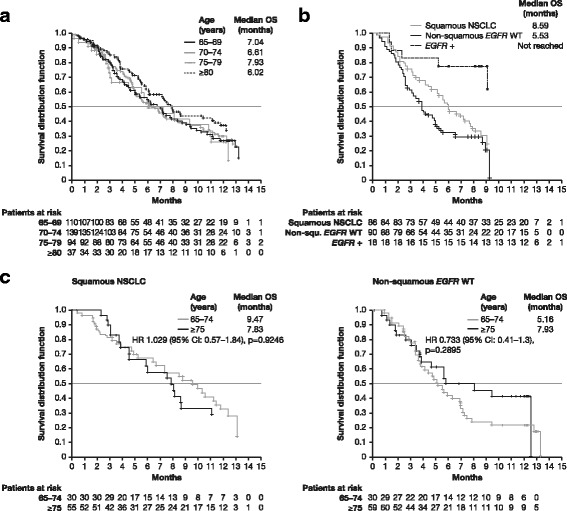
Kaplan-Meier curves on 1-year overall survival in erlotinib-treated patients. **a**) Overall survival in the whole study population according to prespecified age group. **b**) Overall survival in patients with squamous carcinoma, patients with non-squamous *EGFR* wild-type carcinoma and patients with *EGFR* activating mutations. **c)** Overall survival according to age group (< 75 vs ≥75 years) in patients with squamous carcinoma and in patients with non-squamous *EGFR* wild-type carcinoma. CI, confidence interval; *EGFR*, epidermal growth factor receptor gene; HR, hazard ratio; NSCLC, non-small-cell lung cancer; OS, overall survival; WT, wild-type.

Overall, the 1-year OS rate was 31% (95% CI 25–36) with a median OS of 7.1 months (95% CI 6.0–7.9) (Table 2, Fig. 1a). The 1-year PFS rate and the median PFS were 19% (95% CI 15–23) and 3.5 months (95% CI 3.2–4.0), respectively.

The OS curve was significantly affected by gender (*p* = 0.0258), demonstrating 1-year OS rates of 41.8% and 25.4% for females and males, respectively. The log-rank test additionally revealed a significant difference in the OS curves for the subgroup *EGFR* status (*p* = 0.0004, Fig. 1b). In contrast, OS curves were not significantly different between the four age groups (*p* = 0.3436) and the three ECOG PS groups (*p* = 0.5364) (Table 2). Cox regression models with adjustment for single factors showed a significant influence of gender (*p* = 0.027) and *EGFR* status (*p* = 0.001) on OS. Accordingly, females had an almost 30% reduced risk of death compared to males (hazard ratio [HR] 0.717, 95% CI 0.535–0.962). Patients with an *EGFR* mutation had an almost 80% reduced risk of death compared to wild-type patients (HR 0.211, 95% CI 0.083–0.539). In the Cox regression with adjustment for several parameters simultaneously, the association with reduced risk was maintained for positive *EGFR* mutation status (*p* = 0.002, HR 0.177, 95% CI 0.060–0.522). Age did not significantly influence the OS in either analysis (reference 65–74 years, *p* > 0.05).

Patients with squamous NSCLC tended to live longer (median OS: 8.6 months) than patients with documented non-squamous *EGFR* wild-type disease (median OS: 5.5 months) (Table 2; Fig. 1b, c). In addition, patients ≥75 years with non-squamous *EGFR* wild-type carcinoma had a tendency to live longer than their younger counterparts (median OS: 7.93 vs 5.16 months; *p* = 0.2895) (Fig. 1c).

### Symptom control

**Fig. 2:**
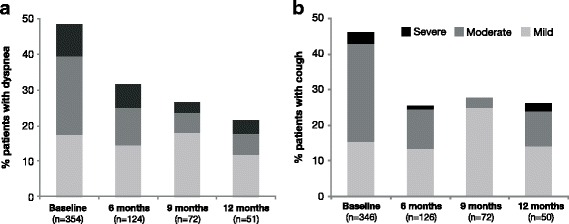
Occurrence of symptoms during the study period. Percentage of patients with mild, moderate and severe dyspnea (**a**) and cough (**b**) at baseline and 6, 9 and 12 months. Percentages were based on patients remaining in the study at the respective timepoints

Symptoms were effectively managed during the observation period. At baseline, 41.6% of patients had cough and 44.4% dyspnea of predominantly mild to moderate intensity. Both symptoms improved at follow-up (Fig. 2). Based on the remaining patients under observation at each visit, only ≤2% of the patients had severe cough at each follow-up visit and severe dyspnea was observed in ≤6.45% of the patients during follow-up (Fig. 2). Post-hoc analysis of *EGFR* wild-type patients showed a similar symptom control compared to the overall population (data not shown).

### Safety and tolerability of erlotinib treatment in elderly patients

During the study, 982 AEs were observed in 296 patients (76.9%) (Table 3). According to the common toxicity criteria for adverse events (CTC), 27.3% of patients had AEs of grade ≥ 3. Serious AEs were reported in 29.1% of patients that led to death in 13.0% of patients. The most commonly reported AEs were rash (45.2%) and diarrhea (22.6%), followed by dyspnea, fatigue, and cough. AEs led to permanent treatment discontinuation in 107 patients (27.8%): Main reasons were rash (26 patients, 6.8%), dyspnea (20 patients, 5.2%), and malignant neoplasm progression (17 patients, 4.4%). The frequency of AEs was not significantly affected by age or *EGFR* mutation status (data not shown). All AEs reported were consistent with those described in the summary of product characteristics [18].

**Table 3:** Overall adverse events (N = 385)

	Patients, *n* (%)
Patients with ≥1 AE	296 (76.9)
Patients with ≥1 AE CTC grade ≥ 3	105 (27.3)
Patients with ≥1 SAE	112 (29.1)
Treatment discontinuations due to AE	107 (27.8)
Most common AEs (frequency ≥ 5%)	
Rash	174 (45.2)
Diarrhea	87 (22.6)
Dyspnea	66 (17.1)
Fatigue	65 (16.9)
Cough	44 (11.4)
Malignant neoplasm progression	31 (8.1)
Decreased appetite	28 (7.3)
Nausea	24 (6.2)
General physical health deterioration	21 (5.5)
Affected system organ class	
Skin	194 (50.4)
Respiratory system	107 (27.8)
Gastrointestinal system	105 (27.3)
General disorders	99 (25.7)
Infections and infestations	44 (11.4)
Neoplasms	40 (10.4)
Metabolic system	35 (9.1)

*AE* adverse event, *CTC* common toxicity criteria, *SAE* serious adverse event

## Discussion

Few data regarding targeted cancer therapy in pretreated elderly NSCLC patients exist. Available study results in elderly patients with advanced NSCLC treated with erlotinib are summarized in Table 4.

**Table 4:** Overview of studies with erlotinib in elderly patients (≥70 years) with advanced NSCLC

Study	Treatment line	Design	Com-parator	Participants	Activating *EGFR* mutation	Outcomes
Jackman et al. (2007) [28]	First line	Open-label, phase 2	None	*N* = 80; 95% Caucasian	9/43 patients tested	• Median OS: 10.9 months (95% CI 7.8–14.6) • 1-year survival rate 46% • Well tolerated: most common AEs were rash (79%) and diarrhea (69%)
Chen et al. (2012) [27]	First line	Open-label, randomized, phase 2	Vino-relbine (V)	*N* = 113; 57 (E) vs. 56 (V); 100% Taiwanese	24/60 patients tested	• Median OS: 17.3 months (E) vs. 22.6 months (V) • Mild toxicities: most frequent treatment-related AEs were rash (64.91%), diarrhea (29.82%), and mouth ulceration (14.04%)
TRUST elderly subgroup [29]	First line	Open-label, phase 4 (subgroup analysis)	None	*N* = 485; 82% Caucasian; 16% Asian	2/18 patients tested	• Median OS: 7.29 months (95% CI 6.27–8.67), non-Asian population: 7.19 months • 1-year survival rate: 36.6% • Disease control rate 79% (compared with 69% for the overall population; *p* < 0.0001) • Good tolerability: only 4% had grade ≥ 3 treatment-related AEs, 7% had treatment-related SAEs (compared with 4% of the overall population)
Stinchcombe et al. (2011) [30]	Any	Open-label, randomized, phase 2	Gem, E + Gem	*N* = 146; 51 (E) vs. 44 (gem) vs. 51 E/gem; US study (included ethnicities not reported)	Unknown	• Median OS with E: 5.8 months (95% CI 3.0–8.3) • Acneiform rash with E: 45% • No significant difference in 6-month PFS, OS and toxicity rates among the groups
POLARSTAR [31]	Any	Open-label,phase 4	None	*N* = 9907^a^; Age-stratified: 7848 (< 75 years), 1911 (75–84 years), 148 (≥85 years); 100% Japanese	Unknown	• Efficacy and incidence of non-hematologic and hematologic toxicities were comparable between age groups
BR.21 Study elderly subgroup [20]	≥Second line	Double-blind, randomized, phase 3 (retrospective subgroup analysis)	Placebo	Erlotinib: 112 (elderly) vs. 376 (young); 9.2% Asian, 90.8% other	15/115 patients tested	• Median OS (elderly vs. young): 7.6 months (elderly) vs. 6.4 months (young); HR 1.02 (95% CI 0.81–1.30) • More overall and severe (grade 3–4) toxicity with erlotinib in elderly vs. younger patients (35% vs 18%; *p* < 0.001)
Keio Lung Oncology Group Study 001 [33]	≥Second line	Phase 2	None	N = 38; 100% Japanese	13/35 tested	• Median OS: 17.3 months (95% CI 13.3–21.3) • Main AE was skin rash (76%)
Lung Oncology Group in Kyushu (LOGiK-0802) [32]	≥Second line	Phase 2	None	*N* = 40^b^; 100% Japanese	10/29 tested	• Median OS: 12.2 months (95% CI 6,1–24,7) • Major toxicities: skin disorders, fatigue, anorexia • 32.5% required dose reduction
ElderTac (present study)	≥Second line	Prospective, non-interventional	None	N = 465^c^; 99.2% Caucasian	18/119 patients tested	• Median OS: 7.1 months • 1-year survival rate: 30.6% • No new safety signals; most frequently reported AEs were rash (45.2%) and diarrhea (22.6%)

*AE* adverse event, *CI* confidence interval, *E* erlotinib, *EGFR* epidermal growth factor receptor gene, *Gem* gemcitabine, *NSCLC* non-small-cell lung cancer, *OS* overall survival, *PFS* progression-free survival, *SAE* serious adverse event

^a^Safety analysis population

^b^Age ≥ 75 years

^c^Age ≥ 65 years

In most studies, patients received erlotinib as first-line treatment [27–29] or the treatment line was not defined [30, 31]. Four studies included exclusively Asian patients [27, 31–33], who are known to have a better outcome with EGFR-TKI treatment compared to Caucasian patients [34]. In the phase-3 trial BR.21, involving 731 patients after progression on ≥1 platinum-based chemotherapy, erlotinib demonstrated prolonged survival [26] and improved QoL compared to placebo [35]. A retrospective subgroup analysis revealed that older (≥70 years) and younger patients had the same survival and QoL benefit, with a somewhat greater toxicity in the elderly [20]. However, the elderly population receiving erlotinib in that study (*n* = 112) [20] was small and a retrospective design involves a greater risk for bias compared with a prospective design.

The ElderTac study was designed to examine the effectiveness and tolerability of erlotinib as a second−/third-line treatment for advanced NSCLC in elderly patients in real life. In accordance with previous findings, females treated with erlotinib lived longer than males [36, 37]. The effectiveness of erlotinib in ElderTac was comparable with that in the BR.21 trial in which median OS/PFS durations of 7.6/3.0 months were observed in elderly patients receiving second- or third-line treatment with erlotinib [20]. Likewise, symptom control – as a surrogate for QoL – was improved in our population, confirming the results of the BR.21 trial [20, 26]. Maintenance of QoL is particularly important in patients with advanced disease receiving second- or third-line treatment. A recent clinical trial demonstrated a similar efficacy for erlotinib and chemotherapy as a second-line treatment for advanced NSCLC in unselected patients and the authors suggested that second-line treatment should be given on patient preference and individual toxicity-risk profiles [24]. However, this recommendation was based on a patient population with a median age of 59 years [24]. In our study, the median age was 72 years and a quarter of patients had an ECOG PS ≥2. Therefore, we have demonstrated that patients with a low performance status, who are not eligible for further chemotherapy, can still benefit from erlotinib. The tolerability of erlotinib in our study was consistent with previous clinical findings in elderly and non-elderly populations, with a tolerable toxicity profile and rash and diarrhea being the most frequently reported AEs [20, 26–28, 30, 38]. No new safety signals were observed. Erlotinib therefore represents a potential palliative treatment for elderly patients with advanced NSCLC.

Based on the mode of action, erlotinib is a more effective treatment for *EGFR*-mutated tumors. As expected, patients with an activating *EGFR* mutation had the greatest benefit from erlotinib treatment, in agreement with previous findings [28, 39]. Nonetheless, consistent with the results of two phase-3 trials [40], our response rates show that *EGFR* wild-type patients can also benefit from erlotinib treatment. A systematic review of the literature and metaanalysis revealed a significant improvement in OS with erlotinib versus other management options in patients with *EGFR* wild-type tumors [41]. In contrast, in the TAILOR and DELTA studies, chemotherapy with docetaxel was more effective than erlotinib for second- or third-line treatment of *EGFR* wild-type patients [42, 43]. However, the populations in TAILOR and DELTA are hardly comparable to the ElderTac population: Patients in TAILOR and DELTA were about six or five years younger and 92.7% or 96.0% of patients had an ECOG PS ≤1, respectively, compared to only 71.2% in ElderTac [42, 43]. Additionally, clinical parameters between the study cohorts in TAILOR were not balanced, as its original concept was not a comparison between erlotinib and docetaxel [42]. In the DELTA study, the subgroup analysis in the unselected population revealed no PFS benefit for docetaxel over erlotinib in patients ≥70 years of age [43], demonstrating that age is an important factor for the treatment decision.

Interestingly, erlotinib-treated patients with squamous tumors tended to live longer than patients with non-squamous *EGFR* wild-type carcinoma, which contradicts previous findings that the prognosis of adenocarcinoma patients is generally better than that of patients with squamous tumors [10]. The finding is unexpected considering the very low *EGFR* mutation rate in squamous tumors but may be explained by *EGFR* gene amplifications frequently found in these tumors [44, 45]. In a phase-4 trial in 1093 patients with metastastic squamous NSCLC, 95% of patients had tumors expressing detectable EGFR and 38% of tumors had a high EGFR expression as confirmed by immunohistochemistry [46]. The LUX-Lung 8 study revealed that the ErbB family blocker afatinib was superior to erlotinib in the treatment of squamous NSCLC [47]. However, the study exclusively included fit patients with an ECOG PS ≤1, and a statistically significant OS benefit for afatinib over erlotinib was only apparent in the subgroup of patients < 65 years of age (HR 0.68, 95% CI 0.55–0.85) but not in patients ≥65 years (HR 0.95, 95% CI 0.76–1.19) [47]. In contrast, our results demonstrate a benefit of erlotinib treatment in older patients (≥65 years) with squamous carcinoma, including unfit patients with an ECOG > 1. A recent case report of a 65-year-old man with *EGFR*-wildtype squamous lung cancer who had an unexpected prolonged response to third-line erlotinib confirms our results [48]. Because genomic alterations have not been comprehensively characterized in squamous tumors, no molecular-targeted therapies have been developed for this NSCLC type so far [45]. Meanwhile, immune checkpoint inhibitors are established first- and/or second-line treatments for NSCLC including squamous tumors [49–52], so that EGFR-TKI will likely move to further therapy lines in patients with squamous *EGFR* wild-type tumors.

A further unexpected result was that older patients with non-squamous *EGFR* wild-type carcinoma (≥75 years) tended to live longer than their younger counterparts. This finding is confirmed by results from a Japanese study with gefitinib in which an age < 75 years was an independent negative factor affecting PFS after EGFR-TKI therapy in patients with advanced NSCLC [53].

Most limitations of our study relate to the nature of a non-interventional trial, especially the lack of a control group and the open-label design. The low rate of *EGFR* mutation testing hampered the comparison of erlotinib effectiveness in a larger group of patients with or without *EGFR* mutations. It, however, reflects the clinical routine in Germany at the time the study was performed, with *EGFR* mutation analysis being done in less than 50% of NSCLC patients [54]. The high rate of treatment discontinuations due to the severely ill patient population might have had an influence on data analysis and interpretation. Furthermore, the results of post-hoc analyses have to be interpreted with caution. Nevertheless, our observational study generated invaluable results for real-life treatment decisions.

## Conclusion

We have demonstrated that erlotinib is a suitable palliative treatment option in further therapy lines for elderly patients with recurrent/advanced NSCLC, especially in patients with an activating *EGFR* mutation and squamous histology. Our results were obtained under real-life conditions and therefore demonstrate effectiveness and tolerability of erlotinib in routine clinical practice.

## Abbreviations

AE: Adverse event
CI: Confidence interval
DCR: Disease control rate
ECOG PS: Eastern Cooperative Oncology Group performance status
EGFR: Epidermal growth factor receptor
*EGFR*: Epidermal growth factor receptor gene
ElderTac: Erlotinib in routine clinical practice in elderly patients with NSCLC
HR: Hazard ratio
NSCLC: Non-small-cell lung cancer
ORR: Objective response rate
OS: Overall survival
PFS: Progression-free survival
PS: Performance state
QoL: Quality of life
TKI: Tyrosine kinase inhibitor
